# Whole genome sequencing reveals complexity in both HPV sequences present and HPV integrations in HPV-positive oropharyngeal squamous cell carcinomas

**DOI:** 10.1186/s12885-019-5536-1

**Published:** 2019-04-11

**Authors:** Ge Gao, Jintu Wang, Jan L. Kasperbauer, Nicole M. Tombers, Fei Teng, Honglan Gou, Yonggang Zhao, Zhenhong Bao, David I. Smith

**Affiliations:** 1 Division of Experimental Pathology, Department of Laboratory Medicine and Pathology, Mayo Clinic, Rochester, MN 55905 USA; 2 BGI-Shenzhen, Shenzhen, China; 3 Division of Otolaryngology, Mayo Clinic, Rochester, MN USA; 4 BGI Americas Corporation, Cambridge, MA USA

**Keywords:** Next generation sequencing, Whole genome sequencing, HPV, OPSCC, HPV integration

## Abstract

**Background:**

High risk human papillomaviruses (HPV) plays important roles in the development of cervical cancer, a number of other anogenital cancer and they are increasingly found in oropharyngeal squamous cell carcinoma (OPSCC), however there has not been comprehensive analysis about the role how these viruses play in the development of OPSCC.

**Methods:**

To characterize the physical status of HPV within OPSCC and to determine the effect this has throughout the host genome, we have performed 30-40X whole genome sequencing (WGS) on the BGI sequencing platform on 34 OPSCCs: 28 of which were HPV positive. We then examined the sequencing data to characterize the HPV copy number and HPV physical status to determine what effect they have on both HPV and human genome structural changes.

**Results:**

WGS determined the HPV copy number across the viral genome. HPV copy number ranged from 1 copy to as high as 150 copies in each individual OPSCC. Independent of HPV copy number, most tumors had either a small or a very large deletion in the viral genome. We discovered that these deletions were the result of either HPV integration into the human genome or HPV-HPV sequence junctions. WGS revealed that ~ 70% of these tumors had HPV integrations within the human genome and HPV integration occurred independent of HPV copy number. Individual HPV integrations were found to be highly disruptive resulting in structural variations and copy number changes at or around the integration sites.

**Conclusions:**

WGS reveals that there is a great complexity in both HPV sequences present and the HPV integrations events in HPV positive OPSCCs tumors. Thus HPV may be playing different roles in the development of different OPSCCs and this further challenge the HPV-driven carcinogenesis model first proposed for cervical cancer.

**Electronic supplementary material:**

The online version of this article (10.1186/s12885-019-5536-1) contains supplementary material, which is available to authorized users.

## Background

High risk human papillomaviruses (HPVs) are known to be involved in the development of cervical cancers, a number of other anogenital cancers and they are increasingly involved in oropharyngeal squamous cell carcinoma (OPSCC) [1–3]. The role(s) that HPV plays in the development of different cancers is not fully elucidated. There is a currently accepted model for HPV-driven carcinogenesis which was derived from several decades of work examining several cervical cancer cell lines and primary tumors. According to the model it is believed that the integration of HPV into the human genome is an important step in carcinogenesis as a frequent site of disruption is the HPV E2 gene which is a transcriptional repressor of the HPV-specific oncogenes: E6 and E7. The viral integration and disruption of the E2 gene would increase expression of the oncogenes E6 and E7 resulting in the degradation of the tumor suppressors p53 and pRB which could eventually lead to cancer development [4, 5]. It is also predicted that the site where the integration occurs within the human genome is random and unimportant [6, 7].

Recent advancements in next generation sequencing (NGS) technologies and their utilization to examine large numbers of primary cervical cancers has revealed that the overall model for HPV-driven carcinogenesis needs to be rethought. An examination of a large number of HPV16 and HPV18-positive cervical cancers has revealed that while most HPV18-positive cervical cancers have HPV integrated somewhere within the human genome, this only occurs in about 75% of HPV16-positive cervical cancers [8]. It was also discovered that there is no preferential disruption of the HPV E2 gene as breakpoints occur throughout the HPV genome in cervical cancer [9]. In addition there are some clustered hot-spots for HPV integrations into the human genome and there are important cancer-related genes at or around HPV integration sites [9]. Hence, important human genes could be contributing to carcinogenesis, and thus the site(s) of integration into the human genome may be more important than initially realized. Whole genome sequencing has revealed the complexity of the HPV18 integration event in the HeLa cell line and this integration occurs within an HPV18-specific hot-spot near the *c-MYC* oncogene. The integration event is also associated with differential amplification of HPV18 sequences, and the nearby human sequences, and the entire size of the region of disruption caused by that integration is over 300 kb [10].

We previously utilized mate-pair next generation sequencing (MP-seq) on the Illumina platform and we only detected HPV integrations into the human genome in 30% of HPV-positive OPSCCs [11]. In this report we utilize the much more comprehensive technique of 30-40X whole genome sequencing (WGS) on the BGI sequencing platform. This has revealed that there is much greater complexity in both the HPV sequences present and the HPV integration events in HPV-positive OPSCC tumors. Our results demonstrate that the different ways that HPV is involved in the development of OPSCCs are still mostly unknown and this has revealed some new features which further challenge the model for HPV-driven carcinogenesis for all cancers with an HPV etiology.

## Methods

### Patients

OPSCC tumors were obtained from patients undergoing surgery in the Department of Otorhinolaryngology at Mayo Clinic from 2006 to 2010. All tissue was snap frozen in liquid nitrogen for long term storage. The study was approved by the Mayo Clinical Institutional Review Board. OPSCC tumor samples were evaluated by a pathologist to confirm that each sample utilized had at least 80% tumor cells on the formalyn-eosin slide. Each patients’ clinical information is listed in Table 1 which includes the patient’s age, gender, tumor anatomic sites, and whether each patient was alive, dead or had tumor recurrence.

**Table 1:** The clinical characteristics of the patients used in this study. Each HPV positive patient has his/her tumor anatomic sites, age range at the surgery, Gender, T-staging and the prognosis status listed in the table. BOT: Base of Tongue. Patients who don’t have their T-stages were marked as blank in the table

	Tumor anatomic site	Age range	T-staging	Live/Recurrence/Dead
468	BOT	50–69	T4	dead
515	Tonsil	30–49	T1	alive
517	BOT	50–69	T2	alive
518	BOT	50–69	T2	dead
522	Tonsil	50–69	T2	alive
523	Tonsil	50–69	T4	alive
525	BOT	50–69	T1	alive
526	Thyroid	50–69		alive (met)
532	Tonsil	≥70		dead
543	Tonsil	50–69	T1	alive
568	BOT	50–69	T1	alive
569	BOT	30–49	T3	alive
601	Tonsil	50–69	T3	alive
614	Tonsil	≥70	T2	alive
624	Tonsil	30–49	T2	alive
655	BOT	≥70	T3	alive (recur)
669	Tonsil	50–69	T4a	alive
670	Tonsil	50–69	T2	alive
676	Tonsil	50–69	T2	alive (recur)
677	Tonsil	30–49	T2	dead
680	Tonsil	50–69	T2	alive (pulmonary mets)
683	Tonsil	≥70	T2	alive
687	BOT	50–69	T2	alive
688	BOT	≥70	T2	alive (lung met)
691	BOT	50–69	T2	alive
711	BOT	30–49	T2	alive
716	BOT	30–49	T3	alive
728	BOT	50–69	T4a	alive

### DNA isolation and HPV detection

Tumor genomic DNA was extracted using the DNeasy Blood & Tissue Kit (Qiagen) according to the manufacturer’s protocol. The presence of HPV16 within each tumor sample was determined by real time PCR using HPV16 specific E6 and E7 primers. The sequence of each primer used was previously reported in Gao et al. [12]. B-actin was used as an internal control.

### Whole genome sequencing

The genomic DNA was quantified by Qubit 3.0 fluorometer (Life Technologies, Paisley, UK), and the integrity was qualified on a 1% agarose gel to make sure the genomic DNA molecular was larger than 23 kb and not substantially degraded. We used the criteria m ≥ 1 μg, c ≥ 12.5 ng/μL and no degradation or partially degraded to define the DNA as “qualified”.

The qualified genomic DNA was randomly fragmented by ultrasound on an Covaris E220 (Covaris, Brighton, UK) according to the manufacturer’s instructions and fragments in the range of 250 bp were obtained by AMPure XP beads (AGENCOURT) after fragment selection. We used MGIEasy DNA Library Preparation Kit V1.2 (MGI Tech Co., Ltd.) for library construction. The end repair of DNA fragments was performed and an “A” base was added at the 3′-end of each strand. Adapters were then ligated to both ends of the end repaired/dA tailed DNA fragments, followed by amplification with ligation-mediated PCR (LM-PCR), then single strand separation and cyclization. Rolling circle amplification (RCA) was performed to produce DNA Nanoballs (DNBs). The DNBs were loaded into the patterned nanoarrays and pair-end reads were determined on the BGISEQ-500 platform for each library and we then ensured that each sample met the average sequencing coverage requirement with at least 30X coverage depth. Sequencing-derived raw image files were processed by BGISEQ-500 base-calling software for base-calling with default parameters and the sequence data of each individual is generated as paired-end reads, which is defined as “raw data” and stored in FASTQ format.

### Sequencing data analysis

1. **Data filtering**

Raw sequence reads were quality controlled using BGI in-house pipeline with multiple filtering steps as follows: 1) removing reads with adapters; 2) removing reads in which unknown bases are more than 10%; and 3) removing reads in which more than 50% of bases are low quality (sequencing quality no more than 10). After filtering, the remaining reads were called “clean reads” and used for downstream bioinformatic analysis.

2) **Mapping and Variant calling**

All clean data of each sample was mapped to the human reference genome (GRCh38/HG38) using the mem algorithm in Burrows-Wheeler Aligner (BWA V0.7.12) [13]. To ensure accurate variant calling, we followed recommended Best Practices for variant analysis with the Genome Analysis Toolkit (GATK, https://www.broadinstitute.org/gatk/guide/best-practices). Local realignment around InDels and base quality score recalibration (BQSR) were performed using GATK, with duplicate reads removed by Picard tools [14–16]. The sequencing depth and coverage for each individual were calculated based on the alignments.

The HaplotypeCaller of GATK(v3.3.0) was used to call both SNPs and InDels simultaneously via local de-novo assembly of haplotypes in a region showing signs of variation. After that, the variant quality score recalibration (VQSR) method, which uses machine learning to identify annotation profiles of variants that are likely to be real, was applied to get high-confident variant calls. The Copy Number Variants (CNVs) were called using the CNVnator v0.2.7 read-depth algorithm, which divides the genome into non-overlapping bins of equal size and uses the count of mapped reads in each bin as the Read-Depth signal [17]. Here we used standard settings and a bin size of 100 bp.

3) **HPV copy number analysis**

After alignment against human reference genomes, we performed computational subtraction of human reads followed by alignment of residual reads to a combined database of human reference genomes and microbial reference genomes (which includes but is not limited to HPV genomes) using BWA software ^12^, resulting in the identification of reads mapping with high confidence to HPV genomes in WGS sample data. SAMtools depth was used to obtain the depth of sequence coverage across the ∼8-kb viral genome based on alignment files in BAM format [18]. To estimate HPV copy numbers per sample, viral genome coverage was divided by human autosomal coverage.

4) **HPV-human integration analysis**

Human reads were subtracted by first mapping reads to a database of human genomes using BWA. The paired-end reads where both ends matched to the human genome were removed in the subtraction process. To identify HPV reads, the remaining reads were aligned with BWA to a combined HPV database that includes multiple HPV reference genomes. An HPV-positive sample was considered integration-positive if there were at least three discordant read pairs and one split read supporting an integration event. Discordant read pairs were defined as having one end of the paired end read mapped to the HPV genome and having its mate pair mapped to the human genome. Split reads were defined as having one end of the paired end read spanning the integration junction and having its mate pair mapped to either the human or HPV genome. To call an integration site, these seven reads together must support integration at the same locus [19].

After HPV reads were obtained, we extracted all pair mates and used BWA to map these paired end reads to a combined database containing the human genome and an HPV genome. Next, soft-clipped reads in the HPV genome were identified and carried forward as potential split reads supporting integration sites. For each split read, we searched for discordant read pairs with one pair mate mapping to the human genome, and we verified that the soft-clipped sequence and the discordant read pairs supported the same location of integration. Read pairs where both mates mapped to the human genome were also used to identify the precise location of the human breakpoint (note that all read pairs that reached this point of the analysis must have had at least one read that had homology with an HPV genome). Finally, if these methods could not allow us to define the precise location of the human breakpoint, we returned to the original human genome alignment and searched for soft-clipped reads in the area of the integration site. Besides, BLASTN and BLAT program was used to double check the integration site [20, 21]. The schematic pipeline for the analysis is included in (Additional file 1 :Figure S1).

5) **HPV-HPV junction analysis**

HPV reads was aligned to the HPV genome using BLASTN 9 to identify the reads spanning different region of HPV genome, indicting the potential HPV-HPV junction events.

### PCR and sanger sequencing validation

To validate suspected HPV integration into the human genome, we designed primers with one of them being derived from the human genome at the potential site of integration and the other against HPV sequences suspected of being near the site of integration within the HPV genome. The primers were designed 200-400 bp away from the detected integration sites based on the WGS paired end reads. To validate the HPV-HPV junction, we designed primers against HPV genome sequences close to the junction sites based on the WGS paired end reads. We performed the end point PCR and each specific PCR product was purified and then Sanger sequenced. The Sanger sequencing was performed by Genewiz LLC.

## Results

### Whole genome sequencing of OPSCCs on the BGI sequencing platform

We performed whole genome sequencing (WGS) on 34 OPSCCs on the BGISEQ-500 platform. 28 of them had HPV16 sequences present within them, while 6 were HPV negative. This was tested by real time PCR with primers targeting the HPV E6 and E7 regions prior to the WGS analysis. The average raw reads obtained for each OPSCC sample was 109, 282.90 Mb and this produced sequencing coverage between a depth of 30-50x for each sample. The clean reads showed a high proportion of sequences with either a Q20 or Q30 sequencing quality. The average number of SNPs detected in each sample was 3,489,583 and 95.65% of the SNPs were annotated in the 1000 Genome Project database. There was an average of 824,792 indels detected in each tumor samples and 54.11% were annotated in the 1000 Genome project database. The summary of the whole genome sequencing data in each tumor sample is shown in the (Additional file 2: Table S1).

After aligning the sequence reads against the human genome, they were then aligned against the HPV16 genome (NC_001526) thus it provided HPV sequence coverage across the entire 7.9 kb viral genome. For the 6 HPV negative tumors, WGS detected very low cross activity with HPV sequence, while the 28 HPV positive tumors had considerably higher HPV sequence reads across the 7.9 kb viral genome. WGS also detected HPV integration events which the sequence reads detected with a portion of a read aligned to the HPV genome and another portion aligned to the human genome. Our main focus in this report was to characterize the HPV copy number present in these samples, to determine the structure of HPV genomes that were present, to ascertain sites of HPV integration into the human genome and then to determine what effect HPV integrations had on human sequences at or near these integration sites.

### HPV copy number varies in OPSCCs and HPV integration is independent of HPV copy number

The average HPV sequencing coverage across the 7.9 kb viral genome ranged from 23.6–6655.4 in the 28 HPV-positive tumors. We then calculated the HPV copy number in each HPV positive OPSCC based upon each tumor’s sequence depth. We specifically chose the house keeping gene β-actin for comparison when we calculated the HPV copy number. There were 5 (18%) tumors that had just a single HPV genome present within them, 12 (43%) tumors that had between 2 and 10 copies of the HPV genome, 6 (21%) tumors had between 10 and 50 copies of the HPV genome, and 5 (18%) tumors with over 50 HPV copies (Table 2).

**Table 2:** HPV16 copy number varies in OPSCC tumors and HPV integration is independent of HPV copy number

HPV16 Sequencing Coverage	20–50	50–500	500–2000	> 2000
HPV16 Copy Number (≈)	1	2–10	10–50	> 50
No.(%) of Samples	5 (18%)	12 (43%)	6 (21%)	5 (18%)
No. of sample with HPV integrations	4	8	4	4

The 30-50x sequence depth obtained with WGS also enabled us to search for putative HPV integration sites within the human genome. We set high stringent filters to rule out false potential integrations where the sequence reads that aligned to the human genome would hit on multiple chromosomes or that mapped within highly repeated sequences in the human genome. A sub-set of the potential HPV integrations were validated by constructing PCR primers across the integrations (with one primer in HPV sequences and the other within human sequences) and the resulting PCR products were Sanger sequenced. WGS detected 20 out of the 28 (~ 71%) HPV16 positive OPSCC tumors which had viral sequences integrated somewhere into the human genome. HPV integrations were found in some of the tumors with only a single copy of the HPV genome, but also in some tumors that had the very high copy number of the HPV genomes. We observed that regardless of HPV copy number, there were tumors with HPV integration and some tumors without integration (Table 2).

### WGS reveals HPV genomes with deletions within them

**Fig. 1:**
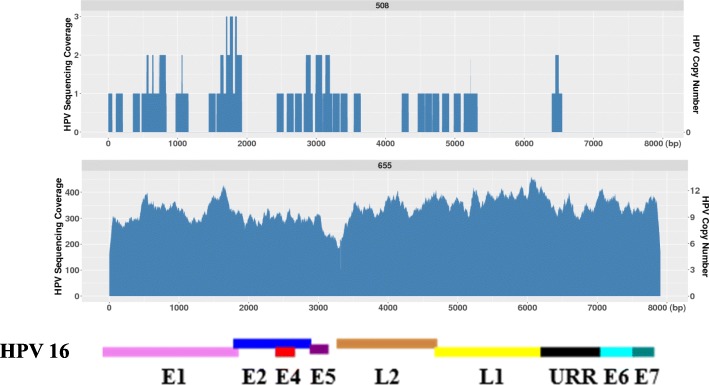
The HPV16 sequencing coverage and the corresponding HPV copy number across the 7.9 kb HPV genome in a HPV negative and a HPV positive tumor. Tumor 508 is HPV negative while tumor 655 is an example of an HPV positive tumor which has approximately ~ 10 HPV copies present. The x- axis represents the 7.9 kb of the HPV 16 genome with the concordant genes listed below; y – axis represents the HPV sequencing read coverage (left) and the corresponding HPV copy number based on the sequencing depth in each tumor (right)

In 28 HPV positive OPSCCs, we found that some of the tumors had intact HPV genomes present while some other tumors had HPV sequences with either small or large deletions within them. We summarize that there were at least four categories representing different types of HPV coverage across the viral genome, indicating the complexity of HPV structures within HPV-positive OPSCCs. In the first category (A): The HPV genome appears intact across the entire 7.9 kb, independent of HPV copy number. In Fig. 1a, tumor 543 only contains a single intact HPV genome, whereas tumor 670 contained ~ 10 intact HPV genomes. In the secondary category (B): There are at least two HPV populations present in a single OPSCC: with one population having the full 7.9 kb HPV 16 genome while the other population only contains partial HPV16 sequences. This category was seen independent of HPV copy number too. In Fig. 1b, tumor 518 has a ~ 2 kb deletion in the E5, L2 and a part of L1 region and this tumor has a HPV copy number around 2; tumor 568 has a ~ 500 bp deletion in the E7 region while its HPV copy number is around 20; tumor 601 has a ~ 6 kb deletions across E6, E6, E1, E2 and a part of the L1 region with an HPV copy number of over 100; while they all kept a portion of the intact 7.9 kb HPV genome. In the third category (C): The third group has what appears to be a single population of HPV genomes, but all of them contain either a small or a very large deletion. This was also independent of HPV copy number. In Fig. 1c, in tumor 669, there is a ~ 5 kb large deletion in the E1-L2 but it is only present as a single HPV copy; in tumor 680, there is a ~ 200 bp deletion in the URR region with an HPV copy number of 2; and in tumor 522, there is a ~ 50 bp deletion in the L2 region but with an HPV copy number of almost 200. In the fourth category (D): HPV copy number varies with small peaks at different regions of the viral genome, with the possibility of much greater HPV complexity as seen in tumors 614 and 468 in Fig. 1d. While in this category, both tumors appeared to have high HPV copy number as their average HPV copy number was ≈30 and ≈100 respectfully however there were certain regions that had an even higher copy number. What is also very interesting about tumor 468 is that even if there are multiple HPV genomes with a variety of different alterations, all of them contain a small deletion near the 3′ end of the HPV genome which encompasses the URR region.

### HPV integration results in partial HPV deletions in the HPV genome

**Fig. 2:**
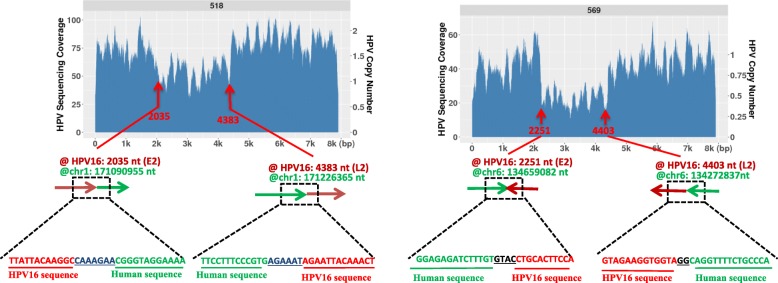
HPV integration into the human genome resulted in the partial deletion within the HPV genome. **a**) In tumor 518, there are HPV-human junction sites detected at the 2035 and 4383 in the HPV16 genome which is exactly where the partial HPV sequences were deleted. **b**). In tumor 569, there are HPV-human junction sites detected at the 2251 and 4403 in the HPV16 genome which is exactly where the partial HPV sequences were deleted. The identified HPV-human junction sequence reads are shown in each figure. The arrow is indicated as 5’to 3′ direction in both human and HPV sequences

We then analyzed those tumors which had deletions in the HPV genome. In tumor 518 which has a low HPV copy number (≈2), we identified 2 HPV-human junctions at nucleotides 2035 and 4383 in the viral genome while those are the sites where the HPV fragment deletions are. In tumor 569 which also has a relatively low HPV copy number (≈1), we identified the HPV-human junction at the 2251 nt and 4403 nt positions in the viral genome while these are also the positions where the deleted viral fragment is located. Figure 2 represents the HPV genome structure and the identified HPV-Human junction reads in tumor 518 and 569. Thus, when HPV exists at low copy, the observed partial viral genome amplification could result from a fragment of HPV integrated and then replicated with the human genome.

### HPV-HPV junctions result in partial HPV deletions in HPV genome

**Fig. 3:**
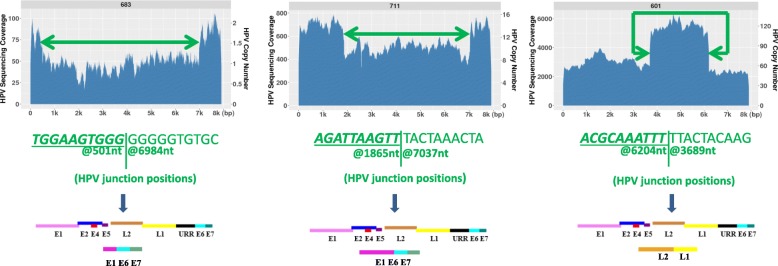
HPV-HPV junction resulted in HPV deletions in the HPV genome. The examples of 3 tumors (683, 711, 601) with different HPV copy number showed two different HPV populations, and the deleted HPV population resulted from HPV-HPV junctions within them. The exact nucleotide positions for the HPV-HPV junctions in the HPV16 genome and the junction sequence reads are listed below. We also represented the map of the potential full HPV and deleted partial HPV genome for each tumor

However, not all the tumors observed with partial deletions (or amplifications) resulted from HPV integration into the human genome. We identified some tumors that had direct HPV-HPV junctions indicative of a deletion in an episomal population of HPV genomes. From 28 HPV positive tumors, excluding the previously described 518 and 569 which have their partial HPV integrated into human genome, there are other 8 tumors which have at least two types of HPV populations, with one that is intact while the other exists as a partially deleted HPV. For example, in the tumor 683, there was a large ~ 6.5 kb deletion observed from 501 to 6884 in the HPV16 genome; in tumor 711, there is a ~ 4 kb deletion observed from 1865 to 7037 in the HPV 16 genome; in tumor 601, the deleted HPV detected is from 6204 to 7906, 1–3689 in the HPV16 genome (Fig. 3). In a total of 8 tumors, we identified that the HPV-HPV junction positions are located exactly where the observed deleted viral regions are. The deleted region could be from 150 nt to 6447 nt. Tumors with these two distinct HPV populations (7.9 kb and the deleted viral genome) are also independent of their copy number. For example, tumor 683 only contains 1–2 HPV copies, 711 contains ~ 12 copies of HPV, while 601 contains ~ 60–80 HPV copies. The rest of the tumors having such features are shown in the (Additional file 3 :Figure S2). The deletion could also occur in any region of the HPV genome. In Table 3, we list each HPV-HPV junction start and end position in the viral genome in these 8 tumors, the deleted viral gene regions, as well as the identified HPV-HPV junction sequence reads. We also want to mention although these tumors have shown deleted HPV genomes co-existing with the full HPV16 genome, some of them also have been detected with HPV integration into the human genome, while some others are found as just episomal copies with two different HPV populations.

**Table 3:** The identified HPV-HPV junction positions and sequence reads in the tumors containing the deleted HPV populations

Tumor	HPV-HPV junction positions in HPV16 genome	Deleted length (nt)	Deleted viral genes	Identified HPV-HPV junction sequence
683	501 --- 6984	6447	E1^a^, E2, E4, E5, L2, L1, URR^a^	***AGTATAGTGGTGGAAGTGGG***GGGGGTGTGCAAACCGTTTT
601	6204 --- 3689	5391	L2^a^, E6, E7, URR, E1, E2, E4, E5	***TTCCTTTAGGACGCAAATTT***TTACTACAAGCAGGATTGAA
711	1865 --- 7037	5172	E1^a^, E2, E4, E5, L2, L1, URR^a^	***GACGTGGTCCAGATTAAGTT***TACTAAACTACAATAATTCA
568	6680 --- 6924	244	URR^a^	***TAAACTTGTACGTTTCCTGC***AGTTCATACATGAACTGTGT
515	1221 --- 7062	5841	E1^a^, E2, E4, E5, L2, L1, URR^a^	***TTGTAAAGGATTGTGCAACA***CTAAGGGCGTAACCGAAATCGGT
532	1091 --- 6897	5806	E1^a^, E2, E4, E5, L2, L1, URR^a^	***TCACAGATGGTACAATGGGC***TTCTAAGGCCAACTAAATGT
676	5852 ---6002	150	L1^a^	***TACATATAAAAATACTAACT***GGAATTTTGGTCTACAACCT
624	6967 --- 1493	2452	URR^a^, E6, E7, E1^a^	***TAAATGTCACCCTAGTTCAT***ACATGAACTGTGTAAAGGTT

^a^indicates that only partial deletion was found in that viral gene

### HPV16 indels and variants / mutations exist in OPSCCs

We also discovered that there are four tumors which have a small fragment deleted in all copies of the HPV sequences present, ranging from 21 nt to 156 nt as we addressed in category B. Tumor 728 has a 21 nt deletion (positions 3516–3536) in L2, tumor 468 has a 34 nt deletion (positions 6844–6978) in URR, tumor 522 has a 156 nt deletion (position 6165–6321) in L2, and tumor 680 has a117nt deletion (position 6165–6321) in L1 region. In addition, we also found that there are sequence variants in different tumors. In tumor 517 there were 14 mismatches variants in its non-coding region; in tumor 687, there are 8 mismatch sequence variants found in E1 and 10 mismatch sequence variants found in E4; in tumor 522, there is a 15 mismatches sequence variant in E4.

### HPV integration is a complex event in OPSCC

Out of the 20 tumors with HPV integrations we found 4 tumors which had virus integrated into just a single chromosome. The remaining 16 had HPV integrated into multiple chromosomes and several of them had HPV integrated in as many as over 6 different chromosomes. As previously described, the integration of HPV into human genome was found independent of the HPV copy number. HPV integration was detected in 4 tumors with a copy number as low as 1, in 7 tumors with copy numbers in the middle range from 2 to 10, in 4 tumors with copy number ranging from 10 to 50, and 3 tumors had an HPV copy number above 50 (Table 2). In Table 4, we listed each tumor with its associated HPV copy number and its integrations into the human chromosomes. We observed that for tumors with a higher HPV copy number, they tended to also have more independent HPV integrations into different chromosomes. All the fiver tumors which had over 20 HPV copies present had the virus integrated into 5 chromosomes or more: they were 614 (≈24 copies), 687 (≈73 copies), 601(≈ 80 copies), 468 (≈83 copies) and 522 (≈140 copies). While in these tumors, the observed HPV copy number also showed the variations with different peaks at different regions as we have previously described in the category D indicating that HPV integration resulted in altered HPV copy number changes as well.

**Table 4:** HPV copy number in each OPSCC with HPV integration events and their integration sites

Tumor	HPV copy number	The chromosome band with HPV integrations
522	143.5	1p33; 1q21.1; 1q24.1; 2p14; 7q32.3; 12q14.1; 17q22
468	83.2	4p11; 10q26.3; 10q21.2; 12q21.2; 17q25.3; 21q11.2
601	79.1	1p34.3; 7q22.1; 8q24.21; 8p23.2; 12p13.31
687	73.4	1p36.11; 2p16.3; 2q36.1; 3q26.1; 8p23.2
614	23.9	2p13.2; 2p23.2; 2p16.1; 2q24.2; 10q23.31
711	13.9	1q23.3; 5q31.1
670	11.9	12q21.33; 12q24.32; 3p11.1
655	9.93	4q34.3
691	9.78	4q13.3; 11q12.3; 11p11.2
515	4.96	1q42.13; 7q11.23; 9q31.3; 11q13.2; 11q13.3
525	4.25	2q14.2; 16q21; 19q13.12; 19q13.31
677	2.62	7p15.3
688	2.41	7q36.3; 7q31.1
683	1.1	Xq26, 5q14.3
680	1.97	8q24.12; 8q24.3
518	1.54	1q24.3; 4q34.3
676	1.06	20q12; 14q32.31; 19q13.43
569	0.78	6q23.2; 2q14.2
669	0.5	12p13.2

We observed that there were several tumors that had multiple HPV-human junctions which were relatively close to one another in the same human chromosome region. We also observed two tumors (518 and 687) that had HPV-human junctions that were considerably further apart. For tumor 518 there were two HPV-human junctions that were 135 Kb apart within chromosomal band 1q24.3, and for tumor 687 there were two human-HPV junctions that were 115 Kb apart within chromosomal band 3q26.1.

All the split reads which contains HPV and human sequence junction are listed in the (Additional file 4: Table S2). Among these, we found that the recurrent integrations occurred in two chromosomal bands: 2q14.2 and 8p23.2. There are no genes identified in or near the HPV integrations sites in the 2q14.2 region, while the HPV integration occurred in 8p23.2 disrupted a very large gene CSMD1 which has been reported as a potential tumor suppressor. It is also interesting to observe that these two chromosome bands are the regions containing multiple HPV integration sites as we previously described. HPV integration frequently occurs in regions where there are no genes, while other HPV integrations occurred within coding genes several of which have previously been suggested to function in tumorigenesis including *CUX1* (7q22.1), *CSMD1* (8p23.2), *EBF3*(10q26.3), *FOXK2*(17q25.3), *CDH11* (16q21), *DNAJB6*(7q36.3), *FOXP2* (7q31.1), *EPHA3*(3p11.1), *PTEN* (10q23.31). In some cases, the HPV integration could result in the disruption of multiple genes.

**Fig. 4:**
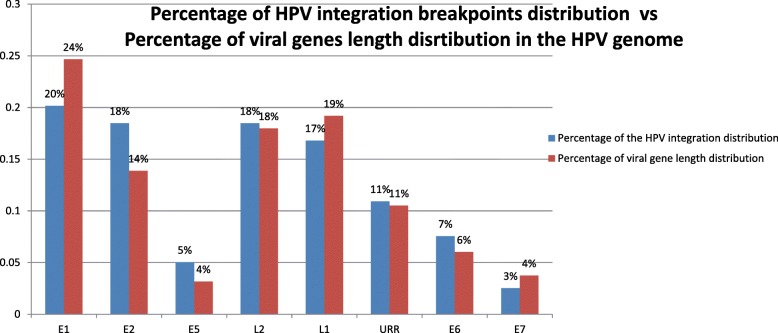
The percentage of the HPV integrations sites in the viral genome is randomly distributed and is proportional to the viral gene length distribution in the HPV genome. The identified human-HPV junctions were compared to the HPV16 genome sequence and integration position was mapped to the HPV16 genome. The blue bars represent the percentage distribution of the HPV integration in each viral gene. The red bars represent the percentage distribution of each of the viral gene’s length

HPV integration were found to occur within any region of the HPV genome including the oncogenes E6 and E7. The distribution of the HPV interruptions breakpoint from all the identified HPV-human junctions in the viral genome is presented in the Fig. 4 which showed HPV integration sites in different regions across the entire HPV genome. Given the different length of the each early and late gene, the number of the breakpoints distributed in the HPV genome is actually proportional to the size of each of the viral genes, suggesting that HPV breakpoints across the vial genome are randomly distributed (Fig. 4). The statistical analysis showed that the percentage of HPV integration breakpoints distributed throughout the HPV genome had a positive correlation to the size of each viral gene in the HPV genome (Additional file 5: Figure S3).

### HPV integration results in structural variations and CNVs at or around the HPV integration sites

**Fig. 5:**
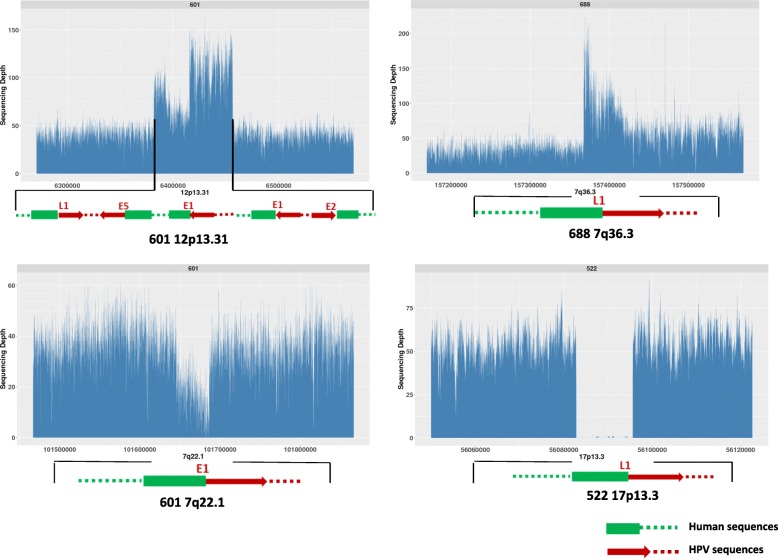
HPV integration resulted in copy number increases or deletions in the human chromosome regions where HPV integration occurred. In tumor 601, HPV integrations occurred in the 12p13.31 which spans a ~ 43 kb region and it also causes 2–3 times copy number changes at the HPV integration sites in the related regions in the human chromosome. In tumor 688, HPV integration in the 7q36.3 results in a 3–4 times copy number increase in the human chromosome regions where HPV integration occurred. In 601 and 522, HPV integration in the 7q22.1 and 17p13.3 lead to the loss of human chromosome sequences

We also examined if there are any human genome structural variations associated with HPV integration events. We found that several of the HPV integration events not only altered HPV sequences at the site of integration but could cause amplification of human sequences at or near the integration sites. In tumor 601, the HPV integration event at chromosome 12p13.31 was also associated with a copy number increase of 43 Kb of human sequences around that integration to 2–3 copies (Fig. 5). Similarly in tumor 688 the HPV integration at 7q36.3 was also associated with a 3–4 times fold increase in human sequences around that integration site. HPV integrations were also found to cause human sequence deletions near the integration sites. For example, we observed that a ~ 100 kb deletion in 17p13.3 in tumor 522 and a ~ 50 kb deletion in 7q22.1 in tumor 601 where HPV integration occurred.

However, not all the HPV integrations caused structural changes in the human genome at the integration site. For example, in tumor 687 which has HPV integrated into 1p36.11 and 4q22.1, there were no significant chromosomal structural changes observed in those integration sites (Additional file 6: Figure S4).

## Discussion

We previously reported that we used Mate-Pair sequencing (MP-Seq) to study the physical status of HPV in OPSCCs and we surprisingly found that there was HPV integration in only ~ 30% of the HPV-positive OPSCCs [11]. MP-Seq is a powerful technique to analyze genomes for gross structural alterations with a limited amount of sequencing [22, 23]. It has even been developed into a clinical test at the Mayo Clinic to analyze structural alterations in cancer. However, WGS provided a much more comprehensive way of analyzing the entire cancer genome at base-pair resolution. At a 30-50x sequence depth, WGS provides detailed information about SNPs, CNVs and other genome alterations. It also reveals the different populations of HPV molecules present in HPV-positive samples. In addition, it also proved to be a more powerful technique to detect HPV integrations as we now have observed that ~ 70% of HPV positive OPSCCs have one or more HPV integrations occurring somewhere within the human genome.

**Fig. 6:**
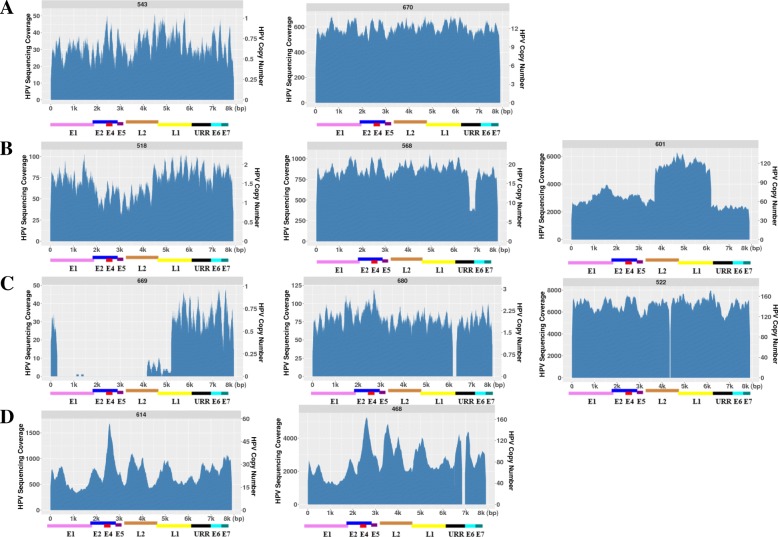
WGS revealed the complexity of the HPV16 sequences present in HPV positive OPSCCs. **a**). OPSCC tumors appeared to have the intact HPV16 sequence across the entire 7.9 kb genome, independent of HPV copy number. Tumor 543 has one HPV copy and tumor 670 has ~ 12 HPV copy. **b**). OPSCC tumors appeared to have at least two HPV populations present in them, independent of HPV copy number. Tumor 518 has ~ 2 HPV copy, tumor 568 has ~ 20 HPV copy and tumor 601 has ~ 100 HPV copy. **c**). OPSCC tumors contains either a small or a very large deletion, independent of HPV copy number. Tumor 669 has ~ 1 HPV copy, tumor 680 has ~ 2 HPV copy and tumor 522 has ~ 150 HPV copy. **d**). OPSCC tumors showed HPV copy number variation with peaks at different regions of the viral genome, mostly seen in the tumors with high HPV copy number

Comparing to other traditional methods in evaluating HPV copy number such as real time PCR for the quantification of one viral region such as L1 or E6/E7, WGS is more powerful in detecting viral copy number across the entire 8 kb viral genome. HPV 16 is known to be the most prevalent HPV subtypes in the development of the OPSCC [12]. For the 34 OPSCC tumors analyzed in this study, prior to WGS analysis, we tested them for the presence of HPV16 sequences using HPV16-specific primers targeting the E6 and E7 region by PCR and 6 tumors were HPV16 negative. Consistent with our findings by real time PCR, WGS did detect very low cross reactivity in these 6 tumors (for example: 508 in Fig. 6) which is most likely caused by human sequences with partial homology to HPV. For the other 28 HPV16 positive tumors, with the exception of tumor 669 which has a ~ 4 kb deletions in the HPV genome, all the other 27 tumors had their viral sequence reads aligned to over 99% of the HPV 16 genome. However we did observe some HPV16 indels and variants/mutations in several cases. A recent review on HPV variants in cervical cancers has defined the HPV linage and sub-linage using empirically defined difference of 1.0–10.0% and 0.5–1.0%, respectively [24]. WGS revealed that their sequences reads had less than 0.5% differences to the reference HPV16 genome indicating that they don’t belong to other HPV subtype and sub-linages based on the current HPV genome variants criteria [24]. Thus WGS is a much more powerful and accurate technique in detecting the HPV copy number, the variations along the HPV genome and the HPV subtypes than other traditional methods.

We found that HPV integration was independent of the HPV copy number in HPV positive OPSCCs. HPV integration was found to occur in tumors that only have a single HPV copy but also in tumors which had more than 100 HPV copies. A previous study with an in vitro cell line (the W12 model) established from cervical intraepithelial neoplasia (CIN I) harboring HPV16 DNA has suggested that HPV integration is accompanied with the loss of episomal HPV copy, which mirrored the classical cervical cancer model [25]. The comprehensive analysis from WGS demonstrates that this may not be the case for OPSCCs as there were several OPSCCs which had HPV integration events but with a very high copy number of HPV sequences. Currently we are not certain if all these HPV copies are present as integrated copies within the human genome, since it’s possible that some of these copies exist in the episomal state. There were some previous reports attempting to determine the relationship between HPV copy number and HPV physical status in cervical cancer and other HPV associated cancers [26, 27]. However the methods that were used in those analyses could not accurately determine the episomal/integrated status. For example, Shukla et al. and Karbalaie Niya et al. used real time PCR to estimate the E2:E6/E7 ratio to determine the HPV physical status [26, 27]. However, there is increasing evidence from our work and others that HPV integration does not preferentially occur in the E2 region [9, 11]. Hence, a comparison of expression between E2 and E6/E7 would not be at all accurate for ascertaining where the HPV genome was disrupted in any integrations. It is also important to realize that 30% of these cancers have developed with just episomal copies of HPV present without any evidence of the HPV integration. Thus, it raises the question as to whether HPV integration is indeed the trigger for cancer development, or whether it occurs after cancer development.

We also investigated if there was any correlation between the HPV copy number/HPV integration status and individual patients’ clinical characteristics, however, due to the limited sample size in this study we didn’t observe any correlation between the viral genome alterations and the aggressiveness of the tumor.

WGS also revealed much greater complexity in HPV positive OPSCCs with respect to the different populations of HPV molecules. We found HPV genomes with either small or very large deletions present among these tumors, independent of HPV copy number. Our results indicated that these deletions could be caused by the integration of HPV into the human genome, but we also found episomal HPV genomes with deletions present within them. What causes these deletions is not known. Our initial results suggested it may have resulted from micro-homology recombination that has occurred in the virus.

There is a previous report from Nulton et al. in which they did a cursory analysis from the TCGA whole genome and RNA sequencing data on around 70 HPV-positive OPSCCs but without any validation [28]. They concluded that the tumors with partial HPV deletions (similar as category C in our results) all have a low viral load and have HPV integrated into the human genome which disrupts the E2 region. However, their conclusion is based on the cervical model assumption that all HPV integrations are accompanied with the loss of the episomal HPV and HPV integration always disrupts the E2 gene which leads to the overexpression of the E6 and E7 oncogenes. We have observed that OPSCC tumors with partial HPV deletions could have a low HPV copy (≈1–2 in tumor 669 and 680), middle range of HPV copy (≈20 in tumor 728) or a high HPV copy (≈150 in tumor 522). The deleted region could be in any portion of the viral genome not necessarily in the E1/E2 region and some of them are associated with HPV integrations but some of them just have episomal copies of HPV present.

Nulton et al. also claimed the presence of the viral-human hybrid episomes in the tumors with partial HPV deletions based on the high DNA copy of both the viral and human genome [28]. However, our results and analysis suggest that this might not be true. First, we examined all the tumors which showed the two different HPV populations with the presence of partially deleted viral genome. For those which have high HPV copy number, we identified that each of those tumors with partial viral deletions have resulted from HPV-HPV junctions where the junction sites are exactly located at ends of the deleted region suggesting that they resulted from partial HPV deletions (Table 3). The high DNA copy at the HPV-human junction sites from the Nulton et al. report more than likely represents integration of viral genomes into the human genome and then dramatic rearrangements at those sites which results in the amplification of both viral and human sequences similar to what has been observed in the HeLa genome [10]. Moreover, our results have provided a more thorough and detailed analysis in terms of the HPV copy number, the different populations of HPV sequences present and how they are related to the HPV integrations. More importantly, we have validated a number of our findings which support the conclusions that we have made.

Previous studies in HPV integrations has indicated that HPV integration was classified into two types: a single HPV genome is integrated into cellular DNA or multiple tandem head-to-tail repeats of the HPV genome are integrated into a single genomic locus [29]. Thus, several models have been proposed as to its oncogenesis: 1). HPV integration disrupts the E1 region by abrogation of E1 replication activities which can induce DNA damage and growth arrest. 2). HPV integration disrupts the viral E2 gene by abrogation of E2 mediated transcriptional repression of the E6/E7 promoter. 3). HPV integration resulted in the generation of viral-host fusion transcripts which harbor the E6 and E7 oncogene which are more stable than the viral E6/E7 transcripts [30]. Our studies and the HPV integration profiling in cervical cancers have suggested that HPV integrations are randomly distributed along the entire viral genome and the integration even could disrupt the E6 and E7 oncogenes. In addition, recent genome analysis of the HeLa cell line has revealed that the entire region disrupted by HPV18 integration spans over 300kbs [10]. But little was previously known about whether or not all, or many, integrations are this disruptive or not. We have now revealed that HPV integrations occurring in the same chromosome region could span over 100 kb as we observed that HPV disrupted 135 kb in 1q24.3 in tumor 518 (2 integration sites identified in 1q24.3), and disrupted 115 kb in 3q26.1in tumor 687 (9 HPV integration sites identified in 3q26.1). HPV integration does not always result in a tandem head to tail but could be very disruptive in terms of both viral sequences and orientations (for example, Fig. 5a in tumor 601). HPV integration could not just dramatically alter HPV sequences but the human sequences at or around the integration sites as we have observed the increased copy number changes in a 43 kb and a 65 kb segment of human sequences in tumor 601 and 518. Due to the short sequence reads (100 bp × 2), we could not assemble the full structure at the sites of the chromosome regions where these HPV integration events occurred. One solution might be to apply the LFR (long fragment read) sequencing technique developed by Complete Genomics so that we could ascertain how HPV integration disrupted the human genome at or around each integration site. Alternatively, long read sequencing technologies might be applied to get a better idea of the structure of the region surrounding individual HPV integration events. Another limitation of the short read WGS performed here is that it’s difficult to know precisely whether a small sequenced portion of the HPV genome present within one of these tumors is derived from an episomal or an integrated HPV genome, unless it contains both HPV and human sequences within the same short fragment.

Some HPV integrations resulted in copy number variations of the human sequences in those regions which could also contribute to HPV induced carcinogenesis. The CNV gain in the 43 kb region in 12p13.31 in 601covers the genes SCNN1A, LTBR and CD27 and CD27 overexpression has been found to be associated with the development of different cancers. The CNV gain in the 7q36.3 in tumor 688 covers the DNAJB6 which has been implicated for its oncogenetic role in promoting cell invasion in colorectal cancer [31].

In addition, we found that tumors with a higher HPV copy number tended to have more independent HPV integrations into different chromosomes suggesting that there might be different numbers of HPV copy integrated into different chromosomes. However we could not determine whether different cells in a single sample had different HPV integration sites or whether every cell had multiple integrations. To address this question, single cell analysis would be an option for future research. In those tumors which had multiple integrations in the same chromosome, the integration sites could be very close to one another and this most likely represents HPV integration resulted rearrangements in both the HPV and human genome. While in some other tumors which had the viral integration sites over 100 kb apart in the same chromosome, we cannot determine whether these represent separate independent HPV integrations or more likely are the result of a single HPV integration across a larger region at the site of integration.

We discovered recurrent HPV integrations occurred in 2q14.2 and 8p23.2. There are no genes identified in or near the HPV integration sites in the 2q14.2 regions, while the HPV integration in 8p23.2 disrupted a very large gene CSMD1 which has been reported as a potential tumor suppressor from different cancers. Previously our mate-pair sequencing also revealed recurrent CSMD1 deletions and inversion in OPSCCs. It was also reported that the alteration of the CSMD1 related genes was associated with a poor overall prognosis in head and neck squamous cell carcinoma (HNSCC) patients [32].

Our results in OPSCC further demonstrate the complexity of this particular type of cancer, as compared to what is known from studies in cervical cancer. How does the presence of just episomal HPV genomes alone could cause cancer development, independent of HPV copy number? Previous next generation sequencing studies have shown that up to 25% of HPV16-positive cervical cancers develop with just episomal copies of HPV present. It has been suggested in those cases that they generally had a high episomal HPV copy which was associated with an increased expression of the viral E6/E7 oncogenes [27]. It has been suggested that in cervical cancer HPV integration is always associated with loss of episomal copies of HPV, but, this is clearly not the case in OPSCC as we have demonstrated. OPSCC has further complications as many of these patients also have a history of smoking and drinking.

In addition, as the classical model for HPV-driven cervical cancer is increasingly being challenged by genome sequence data we must address what is the mechanism whereby HPV promotes the development of cervical cancer and if there are different mechanisms in different cervical cancers. Do any of these mechanisms also play a role in the development of OPSCC? Finally, how does HPV promote cancer in other anogenital sites? Another important, and unanswered question is when during the development of cancers with an HPV etiology does integration occur? We are currently working to determine the structure at and around HPV integrations in OPSCC so that we can begin to address the question about what are the different mechanisms whereby HPV contributes to the formation of many different OPSCCs.

## Conclusion

WGS on the BGI sequencing platform has proved to be a very powerful tool for the characterization of HPV-driven cancers. In addition to providing complete information about SNPs, CNVs and other genomic alterations, WGS has also provided valuable information about the status and structures of the HPV genomes that are present within these cancers, and has revealed that almost 70% of HPV-positive OPSCCs have one, or more, HPV integrations somewhere within the human genome. The copy number of HPV in these cancers ranges from one to 150 copies and HPV integrations occurred independent of HPV copy number. We observe considerable heterogeneity in the HPV genomes that are present and deletions are commonly observed also independent of HPV copy number. In addition, HPV integration can cause human chromosome structural variations that might be associated with alterations of some important tumor related genes, thus additional mechanisms might be involved in HPV-induced cancer development in addition to the known oncogenic role of the HPV viral oncogenes: E6 and E7. Thus, our results further challenge the classical HPV and cervical cancer model and suggest that HPV may be playing different roles in the development of different OPSCCs.

## Additional files

Additional file 1: **Figure S1.** The schematic pipelines for the HPV integration analysis in whole genome sequencing. The figure contains each sequential steps for the analysis of the HPV integration from whole genome sequencing. (DOCX 102 kb)

Additional file 2: **Table S1.** The summary of the whole genome sequencing data in each tumor sample. The table included the total raw reads, the clean reads, the average number of SNPs and indels in each tumor sample. (XLSX 12 kb)

Additional file 3: **Figure S2.** Additional five tumors with HPV deletions within their genome. The figure includes the rest five tumors containing two distinct HPV populations. (DOCX 96 kb)

Additional file 4: **Table S2.** The split reads containing HPV and human sequence junctions. The table listed all the split reads which contains HPV and human sequence junctions. (XLSX 15 kb)

Additional file 5: **Figutre S3.** The correlation chart between the percentage of HPV integration breakpoints distribution and the percentage of viral genes length distribution in the HPV genome. The statistical analysis showed that the percentage of HPV integration breakpoints distributed throughout the HPV genome had a positive correlation to the size of each viral gene in the HPV genome. (DOCX 48 kb)

Additional file 6: **Figure S4.** The tumors without the observed significant chromosomal structural changes in the HPV integration sites. In tumor 687 which HPV integration occurred into the 1p36.11 and 4q22.1, there are no observed significant chromosomal structural changes in those integration sites. (DOCX 34 kb)

## Abbreviations

BWA: Burrows-Wheeler Aligner
CNVs: Copy Number Variants
DNBs: DNA Nanoballs
GATK: Genome Analysis Toolkit
HPV: High risk human papillomaviruses
LM-PCR: ligation-mediated PCR
MP-seq: mate-pair next generation sequencing
NGS: next generation sequencing
OPSCC: oropharyngeal squamous cell carcinoma
RCA: Rolling circle amplification
WGS: whole genome sequencing
