# Supplementary figures and images for: Whole genome sequencing reveals complexity in both HPV sequences present and HPV integrations in HPV-positive oropharyngeal squamous cell carcinomas
Source: BMC Cancer. 2019 Apr 11;19:352. doi: 10.1186/s12885-019-5536-1 (PMC6460540; doi:10.1186/s12885-019-5536-1)

**Additional file 1: Figure S1**


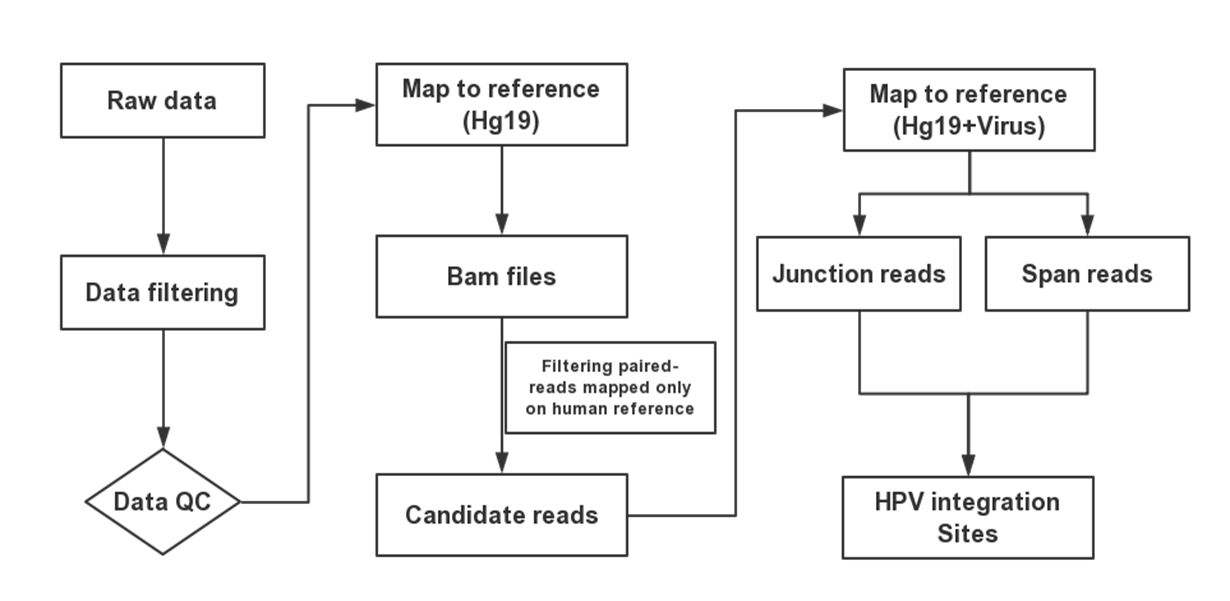

Supplement: Supplementary file 1 — Figure S1. The schematic pipelines for the HPV integration analysis in whole genome sequencing. The figure contains each sequential steps for the analysis of the HPV integration from whole genome sequencing. (DOCX 102 kb) [file 12885_2019_5536_MOESM1_ESM.docx]

**Additional file 3: Figure S2.**

**
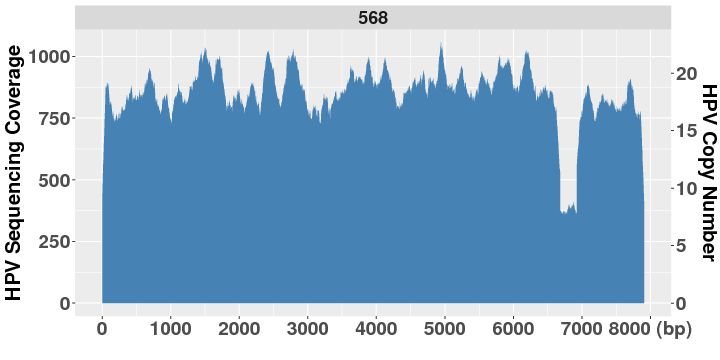

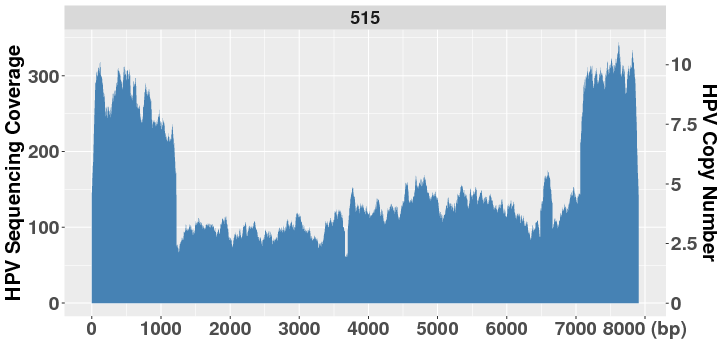

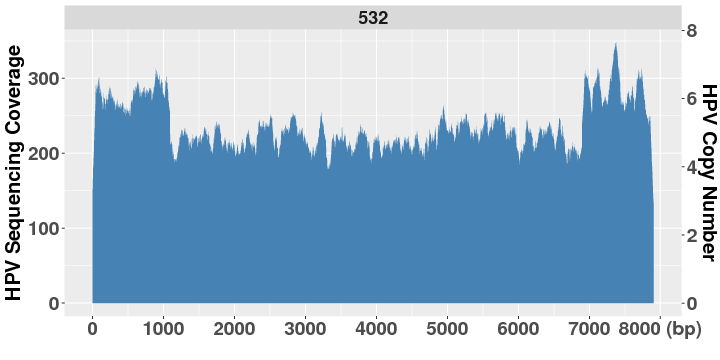

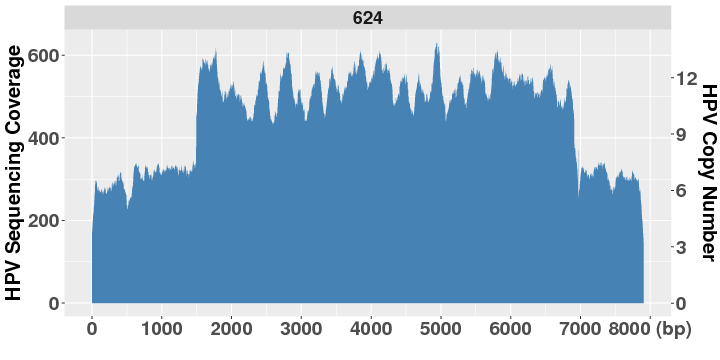

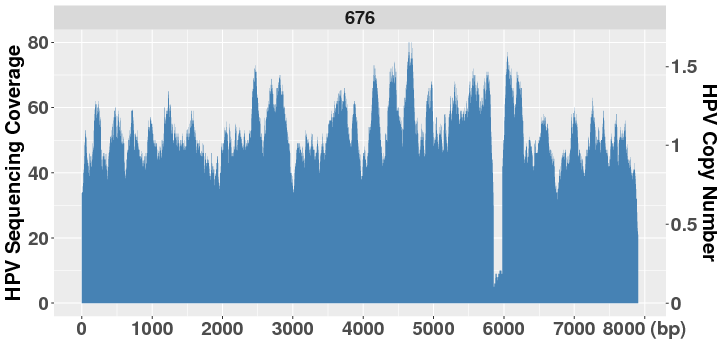
**

**1091**

**6897**

**1221**

**7062**

**6680 6924**

**6967**

**1493**

**5852 6002**

Supplement: Supplementary file 3 — Figure S2. Additional five tumors with HPV deletions within their genome. The figure includes the rest five tumors containing two distinct HPV populations. (DOCX 96 kb) [file 12885_2019_5536_MOESM3_ESM.docx]

**Additional file 6: Figure S4.**


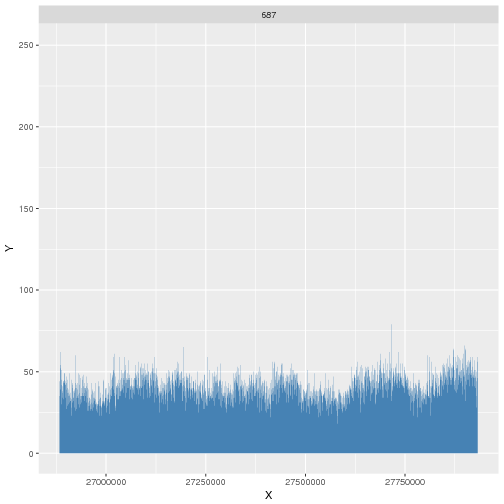

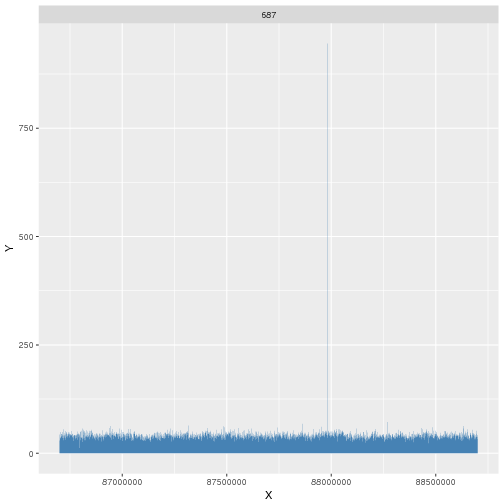


**Chromosome 4q22.1**

**Chromosome 1p36.11**

**Tumor 687**

**Tumor 687**

Supplement: Supplementary file 6 — Figure S4. The tumors without the observed significant chromosomal structural changes in the HPV integration sites. In tumor 687 which HPV integration occurred into the 1p36.11 and 4q22.1, there are no observed significant chromosomal structural changes in those integration sites. (DOCX 34 kb) [file 12885_2019_5536_MOESM6_ESM.docx]
