# Supplementary material for: Whole genome sequencing reveals complexity in both HPV sequences present and HPV integrations in HPV-positive oropharyngeal squamous cell carcinomas
Source: BMC Cancer. 2019 Apr 11;19:352. doi: 10.1186/s12885-019-5536-1 (PMC6460540; doi:10.1186/s12885-019-5536-1)
Supplement: Supplementary file 5 — Figutre S3. The correlation chart between the percentage of HPV integration breakpoints distribution and the percentage of viral genes length distribution in the HPV genome. The statistical analysis showed that the percentage of HPV integration breakpoints distributed throughout the HPV genome had a positive correlation to the size of each viral gene in the HPV genome. (DOCX 48 kb) [file 12885_2019_5536_MOESM5_ESM.docx]

**Additional file 5: Figure S3:**

In tumor 687 which HPV integration occurred into the 1p36.11 and 4q22.1, there are no observed significant chromosomal structural changes in those integration sites.


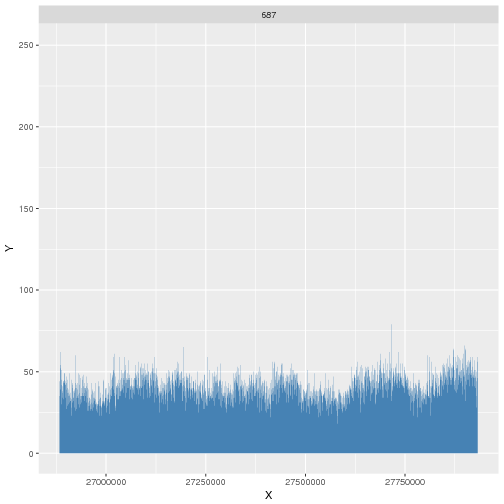

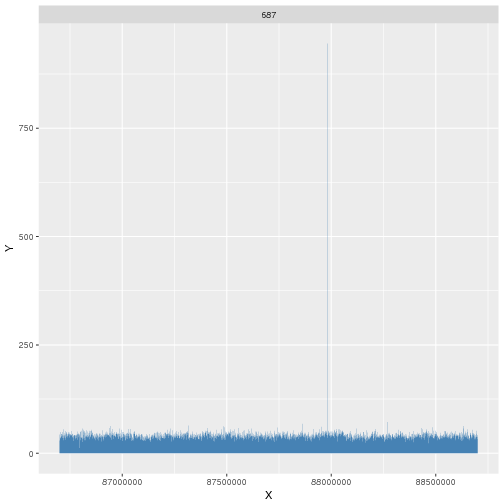


**Chromosome 4q22.1**

**Chromosome 1p36.11**

**Tumor 687**

**Tumor 687**
